# Supplementary material for: Activation of Nrf2/HO-1 by Peptide YD1 Attenuates Inflammatory Symptoms through Suppression of TLR4/MYyD88/NF-κB Signaling Cascade
Source: Int J Mol Sci. 2021 May 13;22(10):5161. doi: 10.3390/ijms22105161 (PMC8152960; doi:10.3390/ijms22105161)
Supplement: Supplementary file 1 [file ijms-22-05161-s001.zip › ijms-1202445-supplementary.pdf]

Supp Figure 1

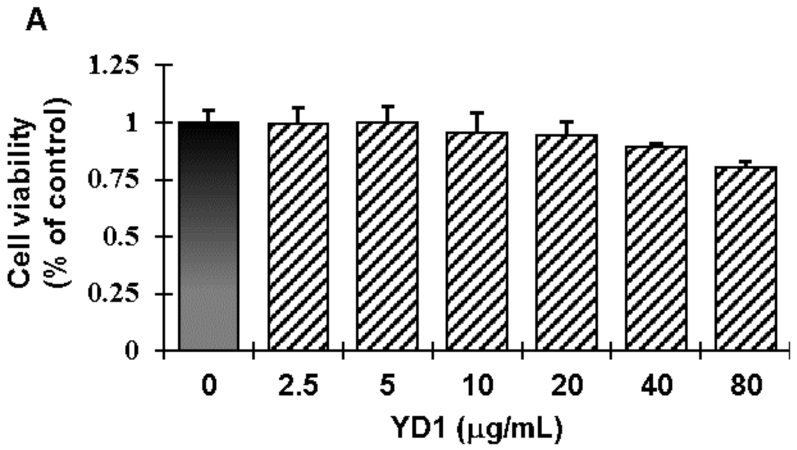


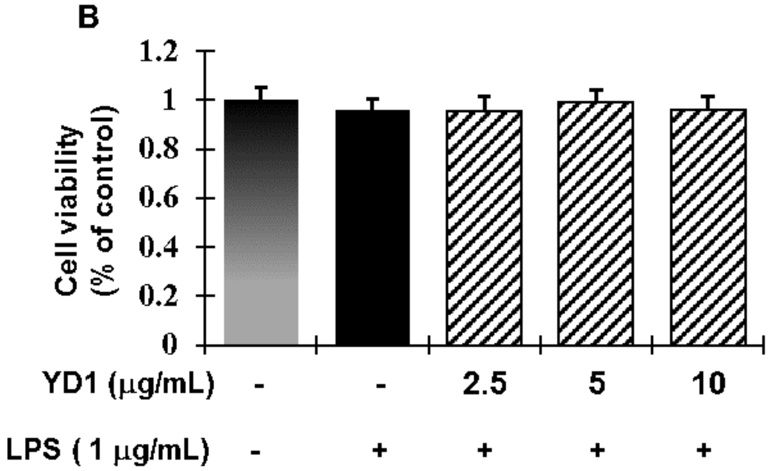

**Table S1: List of the primer sets used in the study**

| *Gene name* | | *Sequences* |
|---|---|---|
| *iNOS* | *forward* | *GGCTGTCAGAGCCTCGTGGC* |
| | *reverse* | *CCCTTCCGAAGTTTCTGGCA* |
| *COX2* | *forward* | *AACACAGCTACGAAAACC* |
| | *reverse* | *CACAGTATGATGTAACAGT* |
| *TNF*$\alpha$ | *forward* | *GGCAGGTCTACTTTGGAGTCA* |
| | *reverse* | *ACATTCGAGGCTCCAGTGAAT* |
| *IL-1*$\beta$ | *forward* | *ATGGCAACTGTTCCTGAACTC* |
| | *reverse* | *CAGGACAGGTATAGATTCTTT* |
| *IL6* | *forward* | *GAGGATACCACTCCCAACAGA* |
| | *reverse* | *AAGTGCATCATCGTTGTTCATA* |
| *Gapdh* | *forward* | *TTGTGATGGGTGTGAACCAC* |
| | *reverse* | *ACACATTGGGGGTAGGAACA* |

**Table S2: List of the primary antibodies used in the study**

| Name | Catalog no. | Company | Antigen | Host |
|---|---|---|---|---|
| Anti-iNOS | MAB9502 | R&D systems | iNOS | Mouse |
| Anti-COX2 | AF4198 | R&D systems | COX2 | Goat |
| Anti-HO-1 | sc-136256 | Santa Cruz Biotechnology, Inc. | HO-1 | Mouse |
| Anti Nrf2 | sc-81342 | Santa Cruz Biotechnology, Inc. | Nrf2 | Mouse |
| Anti-p-IκB-$\alpha$ | sc-8404 | Santa Cruz Biotechnology, Inc. | IκB-$\alpha$ | Mouse |
| Anti-IκB-$\alpha$ | sc-373893 | Santa Cruz Biotechnology, Inc. | IκB-$\alpha$ | Mouse |
| Anti-NF-κB (p65) | BS1254 | Bioworld Technology, Inc. | NF-κB (p65) | Rabbit |
| Anti-Lamin B | BS3547 | Bioworld Technology, Inc. | Lamin B | Rabbit |
| Anti-p-AKT | BS4007 | Bioworld Technology, Inc. | AKT | Rabbit |
| Anti-AKT | BS1008 | Bioworld Technology, Inc. | AKT | Rabbit |
| Anti-TLR4 | BS3489 | Bioworld Technology, Inc. | TLR4 | Rabbit |
| Anti-MyD88 | BS6083 | Bioworld Technology, Inc. | MyD88 | Rabbit |
| Anti-IRAK4 | BS7963 | Bioworld Technology, Inc. | IRAK4 | Rabbit |
| Anti- βactin | Sc-47778 | Santa Cruz Biotechnology, Inc. | β-actin | Mouse |
| Anti-NQO1 | sc-271116 | Santa Cruz Biotechnology, Inc. | NQO1 | Mouse |
